# Clinical skills development in student-run free clinic volunteers: a multi-trait, multi-measure study

**DOI:** 10.1186/s12909-014-0250-9

**Published:** 2014-12-12

**Authors:** Mio Nakamura, David Altshuler, Juliann Binienda

**Affiliations:** Wayne State University School of Medicine, Detroit, MI 48201 USA; Department of Family Medicine and Public Health Sciences, Wayne State University School of Medicine, Detroit, MI 48201 USA

## Abstract

**Background:**

At Wayne State University School of Medicine (WSU SOM), the Robert R. Frank Student Run Free Clinic (SRFC) is one place preclinical students can gain clinical experience. There have been no published studies to date measuring the impact of student-run free clinic (SRFC) volunteerism on clinical skills development in preclinical medical students.

**Methods:**

Surveys were given to first year medical students at WSU SOM at the beginning and end of Year 1 to assess perception of clinical skills, including self-confidence, self-reflection, and professionalism. Scores of the Year 1 Objective Structured Clinical Exam (OSCE) were compared between SRFC volunteers and non-volunteers.

**Results:**

There were a total of 206 (68.2%) and 80 (26.5%) survey responses at the beginning and end of Year 1, respectively. Of the 80 students, 31 (38.7%) volunteered at SRFC during Year 1. Statistically significant differences were found between time points in self-confidence (p < 0.001) in both groups. When looking at self-confidence in skills pertaining to SRFC, the difference between groups was statistically significant (p = 0.032) at both time points. A total of 302 students participated in the Year 1 OSCE, 27 (9%) of which were SRFC volunteers. No statistically significant differences were found between groups for mean score (p = 0.888) and established level of rapport (p = 0.394).

**Conclusions:**

While this study indicated no significant differences in clinical skills in students who volunteer at the SRFC, it is a first step in attempting to measure clinical skill development outside of the structured medical school setting. The findings lend themselves to development of research designs, clinical surveys, and future studies to measure the impact of clinical volunteer opportunities on clinical skills development in future physicians.

## Background

A Student-Run Free Clinic (SRFC) is a service-learning student-driven outreach project in any discipline that strives to enhance the health and well being of a community [1]. Wayne State University School of Medicine (WSU SOM) in Detroit, Michigan opened one such clinic, the Robert R. Frank Student-Run Free Clinic, in 2010. The clinic provides primary care services to the uninsured and underserved. It is administered by medical students at all levels of training and supervised by physician attendings. Roles of the students range from providing primary care, social work, patient education, and laboratory services, as well as maintaining the front desk and pharmacy. Students also have duties outside the clinic such as patient scheduling and follow up, preceptor recruitment, fundraising, and grant writing.

At WSU SOM volunteer recruitment for SRFC begins at the beginning of each school year at the medical school’s organization fair where students are informed of all of the volunteer opportunities and student organizations. Interested students sign up for email notifications of volunteer activities and organizational meetings. Volunteers are also recruited throughout the year through school-wide emails, and students have the opportunity to join SRFC at any time during the school year. Students sign up to volunteer for the many available roles at SRFC.

In the clinic, the primary care team consists of an upper classman (Year 3 or 4) paired with an underclassman (Year 1 or 2). Each team is responsible for interviewing the patient and completing a physical exam. The team will then present the patient to the attending physician. The case is discussed, and the team along with the attending physician sees the patient to conclude the visit. For further management, the patient can be sent to the in-house laboratory, pharmacy, social work services, or patient education, which are all administered by student volunteers. Each volunteer is responsible for documenting the patient encounter in the clinic’s electronic medical records. The documentation of the primary care team in particular is reviewed and signed by the attending physician.

In this way, the SRFC provides a broad range of clinical responsibilities which may imply an increase in clinical knowledge and skills. In addition, the patient population adds a unique experience for the volunteers. However, there have been no published studies measuring the impact of SRFC volunteerism on clinical skills in preclinical medical students. Previous studies have shown that transition from preclinical to clinical years of medical school can be stressful [2]. Students are expected to quickly learn the culture of the hospital while applying basic science knowledge acquired during preclinical years to actual patient care. Clinical exposure during preclinical years of medical school can be beneficial for building self-confidence for the clinical years. Clinical exposure also improves ability to self-reflect, which is important for identifying areas of improvement, integrating new knowledge to existing knowledge, and establishing professional identity [3]. Professionalism is difficult to define; exposure to clinical settings can help establish each student’s own definition of professionalism. All of these skills are valuable components of clinical skills contributing to the creation of a competent physician.

The purpose of this study was to determine if volunteering at a SRFC in the preclinical years of medical school training impacts clinical skill development. The study aimed to specifically measure three related but distinct aspects of clinical skills: self-confidence, self-reflection, and professionalism using validated instruments. The Objective Structured Clinical Exam (OSCE) was also used as a measure of clinical skills.

## Methods

### Surveys

The study was approved by the Institutional Review Board (IRB) at WSU. Four anonymous surveys were distributed to all Year 1 students enrolled in WSU SOM at the beginning (August 2011) and end (April 2012) of the school year: the Experience Questionnaire, Confidence Survey, Groningen Reflection Ability Scale (GRAS) [4], and Pharmacy Professionalism Instrument (PPI) [5]. The Experience Questionnaire asks for participants’ demographic information, as well as baseline clinical experience prior to starting medical school and clinical experience at the end of Year 1, including whether or not they volunteered at the SRFC. The Confidence Survey asks participants to rate their clinical confidence on a 5-point Likert scale. There are 24 items total, 20 of which ask about self-confidence in skills tested on the Year 1 OSCE (see below) and 4 of which are clinical skills that pertain specifically to the SRFC (Table 1). The GRAS is a self-assessment tool validated on 350 first year medical students used to measure a student’s ability to self-reflect. It includes 23 items rated on a 5-point Likert scale. The PPI is a 32-item self-assessment tool rated on a 5-point Likert scale and used to evaluate professionalism. It has been validated on 230 pharmacy students and recent graduates. The tenets of professionalism assessed on the PPI are based on the necessary elements of professionalism emphasized by the American Board of Internal Medicine and is highly applicable to all healthcare professions.

**Table 1:** **Items on the self-confidence survey that pertain to clinical skills practiced at the SRFC**

1. Helping an uninsured patient get medications	3. Counseling patients on lifestyle changes
2. Knowing where to refer uninsured patients for various health care services	4. Asking patients about risky behaviors (i.e. sexual behaviors, drug/alcohol abuse)

### Year 1 objective structured clinical exam (OSCE)

Clinical skills were also measured at the end of Year 1 using the OSCE. The OSCE is based on the SEGUE framework [6] and is required to complete the Year 1 curriculum at WSU SOM. Each student must complete a simulated patient encounter with a standardized patient. In addition to clinical skills, standardized patients were asked to rate the student’s ability to build rapport on a scale of 0 (no rapport), 1 (some rapport), or 2 (excellent rapport).

### Statistical analysis

Responses from all surveys were analyzed quantitatively using IBM SPSS Statistics 19. Results of the Experience Questionnaire were used to divide subjects into two groups by SRFC volunteerism (volunteered at least once during Year 1 or did not volunteer at all during Year 1). For the Confidence Survey, GRAS, and PPI, total scores were calculated in each subject. Then mean and standard error of total scores within each group were calculated. A multivariate analysis of variance (MANOVA) was conducted to compare mean scores in self-confidence, ability to self-reflect, and professionalism (dependent variables) based on group (SRFC volunteer vs. non-volunteer) and time (beginning vs. end of year).

Year 1 OSCE scores were also analyzed quantitatively. Mean score and standard error were calculated for each group (SRFC volunteer vs. non-volunteer). An independent-samples t-test was used to compare mean scores between groups. The frequency of students establishing each of the 3 levels of rapport was identified, and those frequencies were compared between the two groups using a Fischer’s exact test. For all tests, statistical significance was achieved when p < 0.05.

## Results

### Experience Questionnaire

At the completion of surveys at the beginning of Year 1, there were a total of 206 responses out of 302 first year medical students (68.2%). There were 94 (45.4%) male and 112 (54.6%) female students with an average age of 23.6 years. No Year 1 students had volunteered at SRFC at this time. At the completion of the surveys at the end of Year 1, there were 80 (26.5%) responses, 49 (61%) of which were male and 31 (39%) were female with average age of 25.3 years. Of the 80 responses, 49 (61.3%) participants were not volunteers at SRFC during Year 1, and the remaining 31 students (38.7%) had volunteered at SRFC at least once during Year 1. Of these, 13 (43%) subjects volunteered only one time and another 13 (43%) volunteered five or more times.

### Clinical skills surveys

Table 2 shows mean score, standard error, and the 95% confidence interval of each of the three surveys at the beginning vs. end of the year in SRFC volunteers vs. non-volunteers. The analysis of the Confidence Survey was broken down into total score and score of the questions that pertains to SRFC. For the overall score of the Confidence Survey, statistically significant differences were found based on time (beginning vs. end-of-year; p < 0.001), but not based on group (SRFC volunteer vs. non-volunteer; p = 0.544). When looking only at the items on the Confidence Survey that pertain to the SRFC, there was no statistical difference between time (beginning vs. end of the year; p = 0.064), but the difference between groups (SRFC volunteer vs. non-volunteer) was statistically significant (p = 0.032). This indicated that those who volunteered at the SRFC scored higher at both the beginning and end of the year compared to non-volunteers. For the GRAS, comparisons in time (beginning vs. end of the year; p = 0.534) and between groups (SRFC volunteers vs. non-volunteers; p = 0.749) were not statistically significant. This was similarly the case for the PPI (p = 0.265 and p = 0.490, respectively).

**Table 2:** **Results of surveys**

**Volunteered for clinic**	Time points*	**Mean**	**Std. error**	**95% confidence interval**		**P-values**	
				**Lower bound**	**Upper bound**	**Time**	**SRFC volunteerism**
**Confidence Survey (all items)**							
No	1	78.276	2.317	73.615	82.936	<0.001**	0.544
	2	85.724	1.856	81.991	89.458		
Yes	1	77.800	2.790	72.188	83.412		
	2	87.550	2.235	83.054	92.046		
**Confidence Survey (items pertaining to SRFC)**							
No	1	11.759	0.747	10.256	13.261	0.064	0.032**
	2	11.552	0.632	10.281	12.822		
Yes	1	12.600	0.899	10.791	14.409		
	2	15.350	0.761	13.820	16.880		
**Groningen Reflection Ability Scale (GRAS)**							
No	1	84.241	0.914	82.403	86.080	0.534	0.749
	2	84.000	0.982	82.025	85.975		
Yes	1	85.950	1.100	83.736	88.164		
	2	85.200	1.182	82.822	87.578		
**Pharmacy Professionalism Instrument (PPI)**							
No	1	79.103	1.254	76.580	81.627	0.265	0.490
	2	78.724	1.412	75.883	81.565		
Yes	1	79.100	1.510	76.061	82.139		
	2	77.500	1.701	74.079	80.921		

*Time point 1: Beginning of Year 1; Time point 2: End of Year 1.

**Statistically significant findings (P < 0.05).

### OSCE

All 302 first year medical students participated in the Year 1 OSCE, 275 (91%) of which did not volunteer at SRFC and 27 (9%) of which volunteered at SRFC at least once. The average OSCE score of non-volunteers was was 92.6% while that of SRFC volunteers was 92.7% (p = 0.888) (Table 3). The number of subjects establishing a rapport level of 0 was 2; both subjects were non-volunteers (0.7%). The number of subjects establishing rapport level 1 was 41 (15%) within non-volunteers and 2 (7.4%) in SRFC volunteers. The rest of the subjects, 232 (84.4%) of the non-volunteers and 25 (92.6%) of the SRFC volunteers, established rapport level 2. These differences were not statistically significant (p = 0.394).

**Table 3:** **Results of the Year 1 OSCE**

**SRFC volunteer**	**N (%)**	**Mean**	**Std. deviation**	**Std. error mean**	**P-value**
No	275 (91%)	92.606%	4.6161%	0.2784%	0.890
Yes	27 (9%)	92.738%	4.6723%	0.8992%	

## Discussion

The transition from preclinical to clinical years of medical school can be stressful. Students are expected to quickly learn the culture of the hospital while applying basic science knowledge acquired during preclinical years to actual patient care. Clinical exposure during preclinical years of medical school can be beneficial for building self-confidence for the clinical years. Clinical exposure also improves ability to self-reflect, which is important for identifying areas of improvement, integrating new knowledge to existing knowledge, and establishing professional identity. Professionalism is difficult to define; exposure to clinical settings can help establish each student’s own definition of professionalism. All of these skills are valuable in creating a competent physician. This study looked at these three aspects of clinical skills - self-confidence, self-reflection, and professionalism - at the beginning and end of Year 1 in SRFC volunteers and non-volunteers. It also compared scores of the Year 1 OSCE between the two groups.

Statistically significant differences were found in the Confidence Survey only, which showed that overall clinical confidence improves over Year 1. When looking only at clinical skills that are well practiced at SRFC, SRFC volunteers had greater self-confidence than non-volunteers at both the beginning and end of Year 1. This suggests that students who already have some knowledge in providing care to the uninsured and underserved may be more likely to volunteer at SRFC in the first place.

Analysis of the other surveys and the Year 1 OSCE scores did not show any statistically significant findings. This may indicate that young adults who have a well-established sense of self-reflection and professionalism are those that choose to become future physicians. The results of the OSCE scores showed that students perform very well regardless of SRFC volunteerism. The study ultimately found that although the Year 1 curriculum at WSU SOM appears to be adequate to provide students with basic clinical skills expected for the first year of medical school, additional clinical volunteering at SRFC seems to provide a better understanding of providing pertinent psychosocial services needed in treating the uninsured and underserved patient population.

There are several limitations to our study. First, the sample sizes were inconsistent throughout the study at the two survey time points (beginning vs. end of year 1), as well as the OSCE. Participation in the surveys at the end of the school year was significantly less than at the beginning of the year. There was also a discrepancy in the proportion of SRFC volunteers vs. non-volunteers who completed the surveys at the end of Year 1, indicating that SRFC volunteers were more motivated to complete the surveys at the end of the year. Instead of only including subjects who participated in surveys at both time points and the OSCE, which would have made for a small sample size, we decided to analyze the OSCE scores separately to preserve the large sample size that is unique to WSU SOM, the largest single-campus medical school in the country. Nevertheless, the difficulty in encouraging students to participate in a study without compensation was very much apparent. Future studies should attempt to have consistent pre- and post-test subject pools by emphasizing participation. Following these students’ performance in their clinical years of medical school and beyond is an interesting direction for future study.

Second, the PPI was validated for use on pharmacy students only. There are no validated surveys to date specifically for medical students. The PPI was used for the purpose of this study because the tenets of professionalism addressed in the survey were highly applicable to medical students and all healthcare professionals alike. It was created based on the essential traits of professionalism as described by the American Board of Internal Medicine. Nonetheless, future studies should use a scale specifically validated for medical students. The development of such validated instruments provides a focus for future research.

Third, because WSU SOM students historically do well on the Year 1 OSCE, it was difficult to make any meaningful conclusions from the analysis of OSCE scores. Differences may have been more apparent if the patient encounter resembled that of the SRFC. Creating a mock OSCE to model a patient encounter at SRFC may yield interesting results in the future. This will be a difficult task requiring funding, standardized patient training, and student participation.

The rapport building scores on the OSCE were based on a completely subjective scoring system by the standardized patients within an objective exam. The rating of 0, 1, or 2 is an arbitrary level of rapport that is not defined or uniform between each standardized patient. We did not provide specific definitions because it was felt that this better resembles an actual patient encounter. Patients’ definitions of rapport vary depending on the patient characteristics, physician characteristics, and patients’ perspective of their own disease. Even so, a more standardized method to measure rapport, such as patient satisfaction surveys, may provide better direction for future studies.

Lastly, there are many possible confounding variables that affected the outcomes of this study. Students may have had clinical experience prior to starting medical school or volunteered at other clinics throughout Year 1. Future studies should attempt to minimize such biases.

## Conclusions

While this study indicated no significant differences in clinical skills in students who volunteer at the SRFC, it is a first step in attempting to quantitatively measure clinical skill development outside of the structured medical school setting. With approximately 150 student-run free clinics in the country and its number increasing annually, it is necessary to study the impact of such involvement. The findings of this preliminary study lend themselves to the development of research designs, clinical surveys, and future studies to measure the impact of these types of clinical volunteer opportunities on clinical skills development in future physicians.

## Abbreviations

WSU SOM: Wayne State University School of Medicine
SRFC: Student-run free clinic
OSCE: Objective structured clinical exam
GRAS: Groningen Reflective Ability Scale
PPI: Pharmacy Professionalism Instrument
MANOVA: Multivariate analysis of variance
