# Erratum: Clinical skills development in student-run free clinic volunteers: a multi-trait, multi-measure study

**DOI:** 10.1186/s12909-015-0393-3

**Published:** 2015-07-03

**Authors:** Mio Nakamura, David Altshuler, Margit Chadwell, Juliann Binienda

**Affiliations:** 1 Wayne State University School of Medicine, Detroit, 48201 MI USA; 2 Department of Family Medicine and Public Health Sciences, Wayne State University School of Medicine, Detroit, 48201 MI USA

## Erratum

After publication of this work [1], we noted that we inadvertently failed to include the complete list of all co-authors. The full list of authors and the corrected Authors Contributions section are reported below. We apologize for any inconvenience this oversight may have caused.

## Corrected authors’ list

Mio Nakamura^1*^, David Altshuler^1^, Margit Chadwell^2^, Juliann Binienda^2^.

## Corrected authors’ contributions

MN made substantial contributions to conception and design, acquisition of data, analysis and interpretation of data, and drafted the manuscript. DA made substantial contributions to conception and design, acquisition of data, analysis and interpretation of data. MC made substantial contributions to the inception of the study, revision of the manuscript, and is Medical Director of the student-run free clinic. JB made substantial contributions to conception and design, analysis and interpretation of data, and revised the manuscript. All authors read and approved the final manuscript.
